# Prevalence and Impact of Apolipoprotein E7 on LDL Cholesterol Among Patients With Familial Hypercholesterolemia

**DOI:** 10.3389/fcvm.2021.625852

**Published:** 2021-04-13

**Authors:** Hayato Tada, Kan Yamagami, Nobuko Kojima, Junichi Shibayama, Tetsuo Nishikawa, Hirofumi Okada, Akihiro Nomura, Soichiro Usui, Kenji Sakata, Masayuki Takamura, Masa-aki Kawashiri

**Affiliations:** Department of Cardiovascular Medicine, Kanazawa University Graduate School of Medical Sciences, Kanazawa, Japan

**Keywords:** APOE, familial hypercholesterolemia, PCSK9, LDLR, LDL-C

## Abstract

**Background:** It has been suggested that a rare mutant apolipoprotein E7, APOE7 (p.Glu262Lys, p.Glu263Lys), has been identified to be associated with hyperlipoproteinemia in the general population. Moreover, its prevalence has been shown to be 0.005–0.06%. However, there are no prior data regarding its prevalence and impact on serum lipids in patients with familial hypercholesterolemia (FH).

**Methods:** We recruited 1,138 patients with clinically diagnosed FH [mean age = 48, men = 512, median low-density lipoprotein (LDL) cholesterol = 231 mg/dl]. The coding regions of three FH genes (*LDLR, APOB*, and *PCSK9*) and apolipoprotein E (*APOE*) gene were sequenced. We investigated the prevalence and impact of APOE7 mutant on serum lipid levels in patients with FH.

**Results:** We identified 29 patients (2.5 %) with a mutant APOE7 (heterozygote), which is apparently much higher than that of the general population. Moreover, when we focus on those without FH mutation (*n* = 540), we identified 21 patients (3.9 %) with a mutant APOE7. Patients with a mutant APOE7 exhibited significantly higher median LDL cholesterol and triglyceride levels compared with those without this rare mutant (249 vs. 218 mg/dl, *p* < 0.05, 216 vs. 164 mg/dl, *p* < 0.05, respectively). Moreover, LDL cholesterol levels in the APOE7-oligogenic FH individuals, with a pathogenic mutation in FH genes and APOE7 mutant, were significantly higher than that in monogenic FH patients (265 vs. 245 mg/dl, *p* < 0.05).

**Conclusion:** We identified more patients with a mutant APOE7 than expected among those diagnosed with FH clinically, especially among those without FH-causing mutation. This implies a mutant APOE7 may be one of the causes FH, especially among those without FH mutations.

## Introduction

Patients with familial hypercholesterolemia (FH), caused by genetic mutations in low-density lipoprotein (LDL)-associated genes (FH genes), are typically exhibiting extreme hyper-LDL cholesterolemia, tendinous xanthomas, and premature atherosclerotic cardiovascular disease (ASCVD) (1, 2). Even though it is a genetic disorder, at least a part of clinically diagnosed FH patients do not have any causative mutations in FH genes even with the comprehensive genetic analyses (3). It is possible that novel genes are contributing to this disease, or accumulation of LDL-associated common genetic variations may have an impact on this disease. In contrast, apolipoprotein E (APOE) has been shown to interact with LDL receptor (LDLR) (4). Moreover, the *APOE* is highly polymorphic: APOE-ε2 (cys112/cys158), APOE-ε3 (cys112/arg158), and APOE-ε4 (arg112/arg158) (5, 6). These common polymorphisms have been associated with LDL cholesterol levels (7). In addition to these common polymorphisms, a rare mutant of APOE7 (p.Glu262Lys, p.Glu263Lys) has been identified to be associated with dyslipidemia in the general population. It has been shown that additional lysine residues of apoE7 were associated with reduced binding affinity to LDLR and increased affinity to heparin; both of those features appear to lead to their atherogenic lipoprotein profile (8). Moreover, its prevalence has been shown to be 0.005–0.06% (9–12). According to gnomAD browser, the allele frequency of the e7 variant is 0.002698 in East Asians, and it is not found among other ethnicities (13). Another study showed that the LDLR binding activity of APOE7 has been reduced to 23% *in vitro* (14). Moreover, interesting cases with a mutant APOE7 exhibit phenocopy of FH, including extremely high LDL cholesterol level and Achilles tendon thickness (15).

Recently, we showed that loss-of function mutations in ATP-binding cassette subfamily G member 5 (*ABCG5*) or ATP-binding cassette subfamily G member 8 (*ABCG8*) considered as causes of recessive disorder contribute to mimicking or exacerbating the phenotype of FH (16, 17). Considering these backgrounds, we tried to investigate whether a rare mutant of APOE7 is also contributing to mimic and/or exacerbate of the phenotype of FH.

## Methods

### Study Population

In this study, we included 1,262 clinically diagnosed FH patients (18) at our institution from 2014 to 2020. We used the clinical diagnostic criteria of FH determined by Japan Atherosclerosis Society. A total of 121 subjects were removed due to missing data. Moreover, three individuals with double mutations were excluded. Accordingly, 1,138 subjects [male, 45%; female, 55%; mean age = 48 years; ASCVD = 211 (19%)] were finally included in this study.

### Genetic Analysis

**Figure 1:**
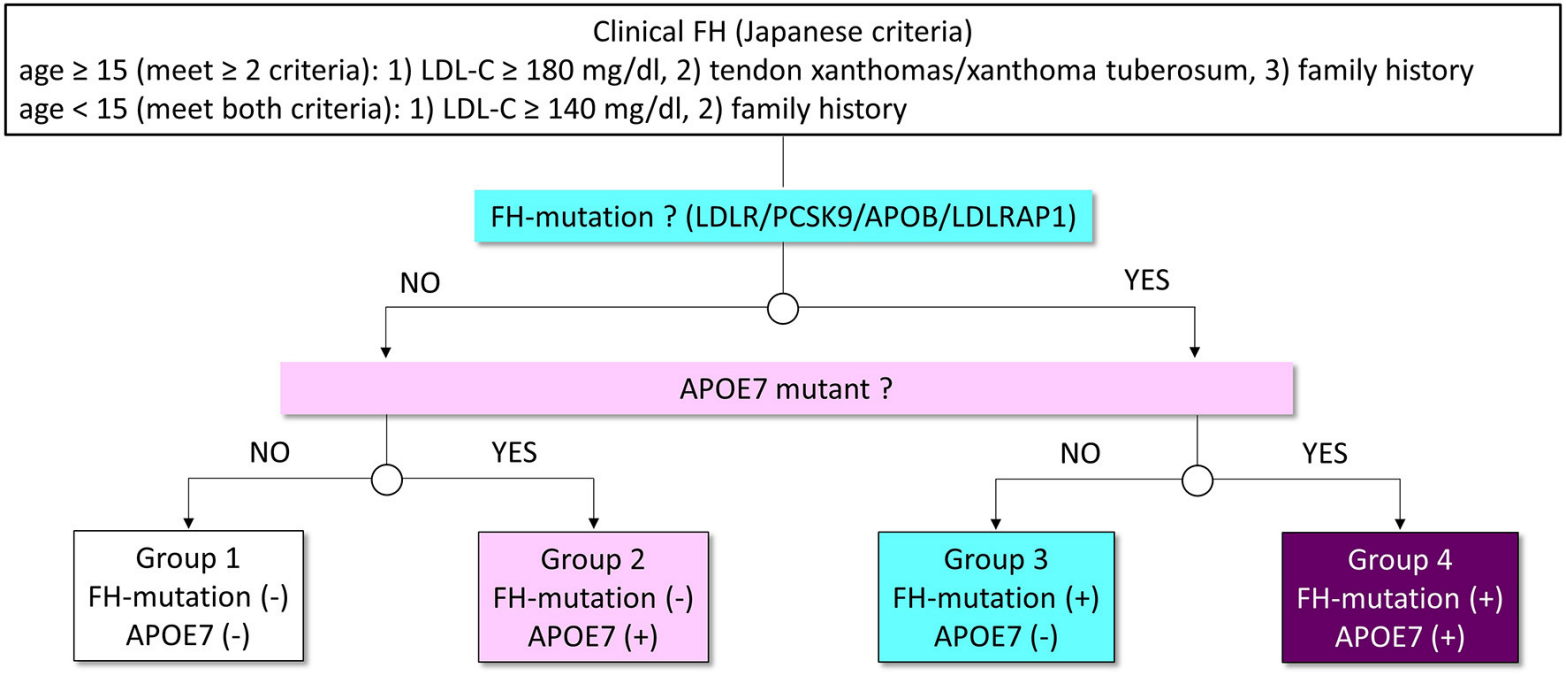
Analytic scheme in this study. Patients with FH were classified into four groups based on the status of FH mutation and APOE7 mutant (Groups 1–4). White indicates patients without mutations. Pink indicates patients with APOE7 mutant. Light blue indicates patients with a mutation in FH genes. Purple indicates patients with a mutation in FH genes and APOE7 mutant.

The coding region of 21 genes (*ABCA1, ABCG5, ABCG8, ANGPTL3, APOA1, APOB, APOC2, APOC3, APOA5, APOE, CETP, GPIHBP1, LCAT, LDLR, LDLRAP1, LIPG, LMF1, LPL, MTTP, PCSK9*, and *SAR1B*) associated with Mendelian lipid diseases, including three FH genes (*LDLR, APOB*, and *PCSK9*) and *APOE* gene were sequenced. In this study, we defined pathogenic mutations as those raising LDL cholesterol. The pathogenicity of the variants was assessed through the allele frequency in the Asian population of The Exome Aggregation Consortium (19), computational pathogenicity prediction tools, and ClinVar. The variants with allele frequency <5% are considered as rare. In addition, we determined variants as pathogenic using the standard American5College of Medical Genetics (ACMG) criteria. The subjects were divided based on the presence of FH mutation and APOE7 mutant (heterozygote; Figure 1).

### Ethical Considerations

The Institutional Review Board of Kanazawa University approved this study. All procedures were in accordance with the ethical standards of the committee on human experimentation and Declaration of Helsinki of 1975, revised in 2008. Informed consent was given to us from all of the study participants in this study.

### Biochemical Test

All blood tests were performed at fasting state. The serum lipids, including total cholesterol, triglycerides, and high-density lipoprotein cholesterol, were assessed enzymatically (20). LDL cholesterol levels were calculated using the Friedewald formula if the triglyceride levels were <400 mg/dl; otherwise, they were determined directly. All of the data presented in this study were assessed at the point without any lipid-lowering therapies.

### Clinical Assessments

We defined hypertension as systolic blood pressure 140 mmHg or greater, diastolic blood pressure of 90 mmHg or greater, or under the antihypertensive treatment. We used the definition of diabetes determined by the Japan Diabetes Society (21). Coronary artery disease (CAD) was defined as any incident associated with coronary artery stenosis assessed through angiogram and/or computed tomography (22).

### Statistical Analysis

Fisher's exact test or chi-square test was used for categorical variables. Continuous variables with a normal distribution were presented as mean ± standard deviation. Median values and interquartile ranges (IQRs) were presented for those exhibiting non-normal distribution. Student's *t*-test was used for continuous variables. Median values were compared using nonparametric Wilcoxon Mann–Whitney rank sum test or chi-square test with Fisher's *post-hoc* test. Multivariable linear analyses, including age and sex, were used to assess the association between genetic variations and LDL cholesterol. Kruskal–Wallis one-way analysis of variance followed by Tukey *post-hoc* test were used to test the differences among the groups. All of the statistical analyses were conducted with R statistical software, and *p* < 0.05 were considered significant statistically.

## Results

### Characteristics

The characteristics are presented in Table 1. The mean age was 48 years, and 211 (19%) of the patients had CAD. There were 590 patients with monogenic FH with a pathogenic mutation in one of the FH genes without APOE7 mutant (52%, monogenic FH, group 3), whereas 519 patients did not have FH mutations or APOE7 mutant (46%, mutation negative, group 1). Twenty-one patients had APOE7 mutant but not FH genes (2%, APOE7 mutant carriers, group 2). We identified eight patients with pathogenic mutations in FH genes as well as in APOE7 mutant (0.7%, APOE7-oligogenic FH, group 4).

**Table 1:** Characteristics of the patients.

	**All**	**Classification**				
**Variable**	**(*n* = 1,138)**	**Group 1 (*n* = 519)**	**Group 2 (*n* = 21)**	**Group 3 (*n* = 590)**	**Group 4 (*n* = 8)**	***p*-value**
		**No mutation**	**APOE7 mutant**	**Monogenic FH**	**Oligogenic FH (monogenic FH + APOE7 mutant)**	
Age (years)	48 ± 20	53 ± 18	44 ± 21	40 ± 19	42 ± 21	<1 × 10^−16^
Male	512 (45%)	236 (45%)	11 (52%)	260 (44%)	5 (63%)	0.18
Hypertension	213 (19%)	117 (23%)	5 (24%)	91 (15%)	0 (0%)	0.012
Diabetes	69 (6%)	33 (6%)	5 (24%)	31 (5%)	0 (0%)	6.9 × 10^−4^
Smoking	221 (19%)	124 (24%)	7 (33%)	89 (15%)	1 (13%)	0.14
Total cholesterol (mg/dl)	306 [279–366]	303 [271–327]	343 [277–389]	327 [264–391]	355 [298–398]	4.6 × 10^−5^
Triglyceride (mg/dl)	169 [88–204]	164 [81–182]	216 [94–256]	145 [73–199]	188 [87-314]	8.9 × 10^−4^
HDL cholesterol (mg/dl)	52 [42–64]	52 [43–65]	51 [44–61]	52 [44–63]	51 [46–62]	0.23
LDL cholesterol (mg/dl)	231 [191–271]	218 [189–243]	249 [196–277]	245 [201–281]	265 [216–319]	7.2 × 10^−6^
RLP cholesterol (mg/dl)	7.4 [2.5–9.8]	7.3 [2.4–9.6]	12.3 [5.8–21.5]	7.2 [2.6–8.8]	11.4 [2.5–19.0]	1.6 × 10^−4^
CAD	211 (19%)	102 (20%)	2 (10%)	105 (18%)	2 (25%)	0.026

*FH, familial hypercholesterolemia; CAD, coronary artery disease; RLP, remnant-like particle*.

### Impact of APOE7 (Heterozygote) Mutant on LDL Cholesterol

**Figure 2:**
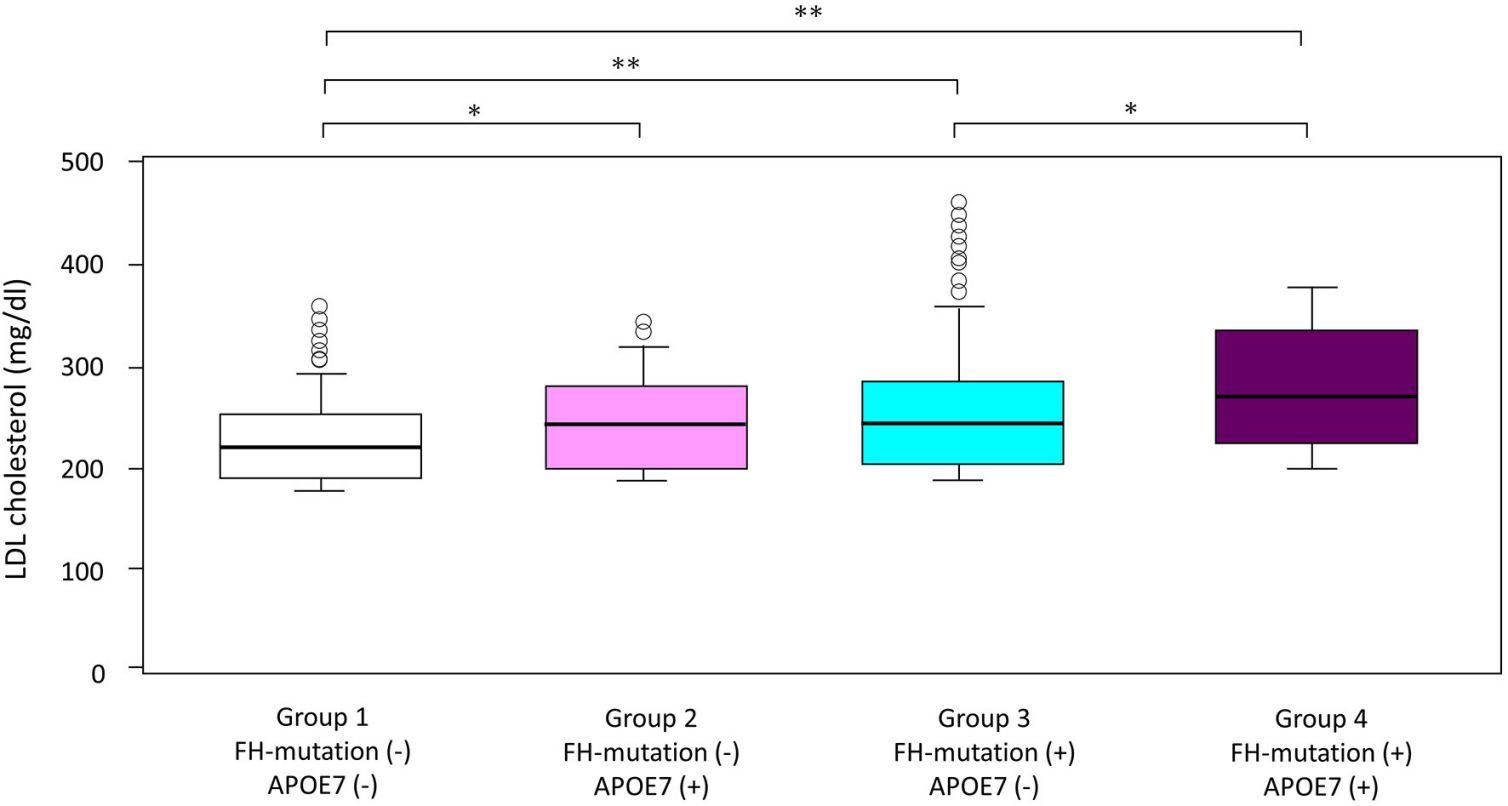
Impact of FH gene and APOE7 on LDL cholesterol. Boxplots are showing LDL cholesterol. ^*^p < 0.05 and ^**^p < 0.001. Analyses of variance followed by Tukey *post-hoc* test were used to test the differences among the groups.

LDL cholesterol levels in patients with APOE7-oligogenic FH, who had pathogenic mutation in FH genes as well as APOE7 mutant, were significantly higher than those in patients with monogenic FH with a single pathogenic mutation in FH gene (265 vs. 245 mg/dl; Table 1 and Figure 2). Moreover, LDL cholesterol level in patients with monogenic FH was significantly higher than those without any mutations (group 1) (245 vs. 218 mg/dl; Table 1 and Figure 2). Under these conditions, LDL cholesterol level of APOE7 mutant carriers (group 2) was significantly higher than that of the patients without any mutations (group 1) (249 vs. 218 mg/dl) (Table 1 and Figure 2).

### Impact of APOE7 Mutant on Triglycerides and Remnant-Like Particle Cholesterol

**Figure 3:**
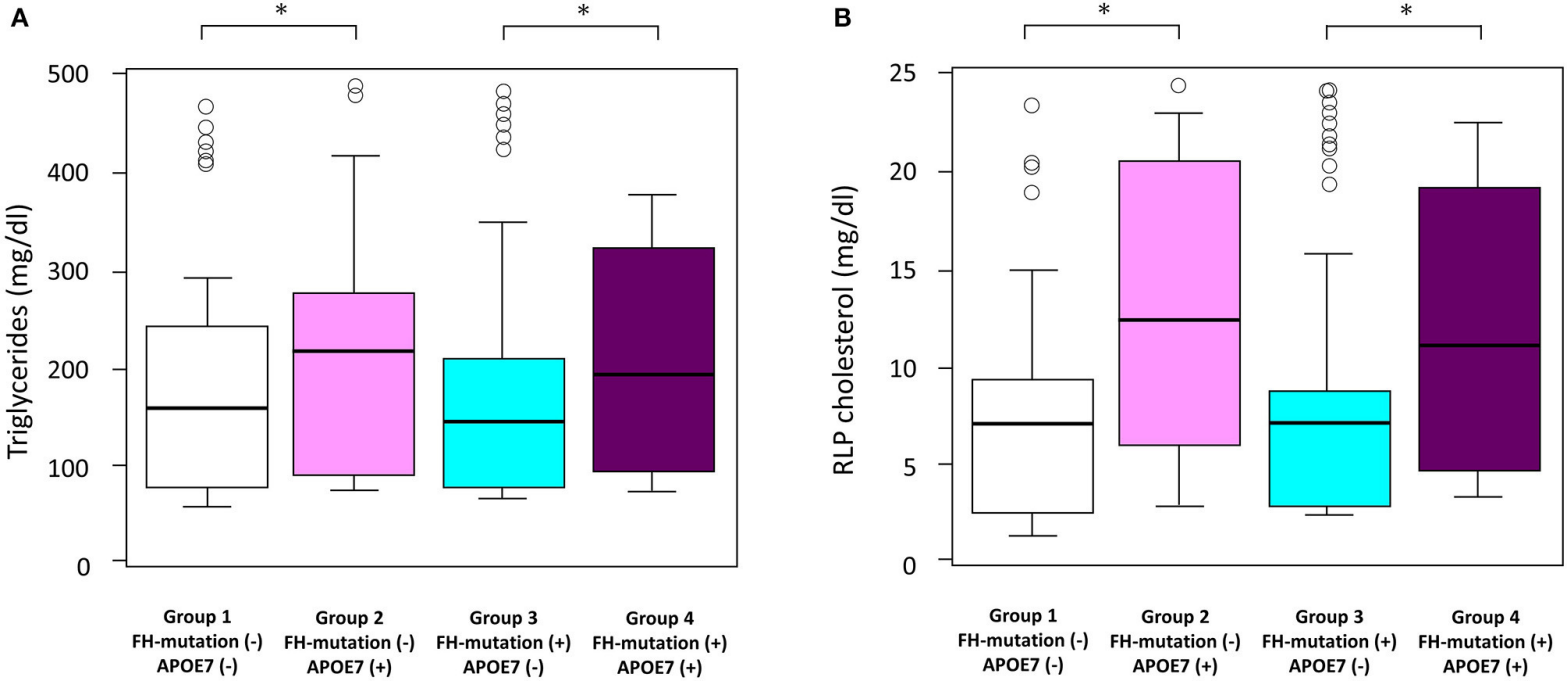
Impact of FH gene and APOE7 on **(A)** triglyceride and **(B)** RLP cholesterol levels. Boxplots are showing triglyceride and RLP cholesterol. ^#x0002A;^*p* < 0.05. Analyses of variance followed by Tukey *post-hoc* test were used to test the differences among the groups.

In addition to LDL cholesterol, triglyceride levels in the APOE7 mutant group (group 2) were significantly higher than those without any mutations (group 1) (216 vs. 164 mg/dl; Table 1 and Figure 3A). Additionally, remnant-like particle cholesterol levels in the APOE7 mutant group (group 2) were significantly higher than those without any mutations (group 1) (12.3 vs. 7.3 mg/dl; Table 1 and Figure 3B).

### Associations Between LDL-Associated Genetic Mutations and LDL Cholesterol

Multivariate linear analyses revealed that one mutation in the FH genes increased LDL cholesterol level by 46 mg/dl (95% CI, 21–51; *p* = 2.3 × 10^−12^; Table 2), while an APOE7 mutant elevated LDL cholesterol by 31 mg/dl (95% CI, 6–45; *p* = 7.4 × 10^−4^; Table 2). Moreover, we found that one protein-truncating mutation had much larger effect on LDL cholesterol than one missense mutation in *LDLR* or *PCSK9* (Table 2).

**Table 2:** Mutation type and its association with LDL cholesterol.

**Variable**	**Effect (mg/dl)**	**95% CI**	***p*-value**
Mutation (all FH genes) [*n* = 598]	46	21–51	2.3 × 10^−12^
Protein truncating mutation (*LDLR*) [*n* = 305]	50	29–71	<1 × 10^−16^
Missense mutation (*LDLR*) [*n* = 214]	38	23–44	4.4 × 10^−7^
Missense mutation (*PCSK9*) [*n* = 79]	28	10–39	3.9 × 10^−5^
APOE7 mutant [*n* = 29]	31	6–45	7.4 × 10^−4^

*FH, familial hypercholesterolemia; LDLR, LDL receptor; PCSK9, proprotein convertase subtilisin/kexin type 9; CI, confidence interval*.

*Multivariable linear analyses, including age and sex, were used to assess the association between genetic variations and LDL cholesterol*.

## Discussion

We aimed to assess the impact of a rare mutant APOE7 on serum lipids in clinically diagnosed FH patients. Among patients without any FH mutations, 21 (4%) had APOE7 mutant (heterozygote). We also identified eight individuals with a FH mutation and APOE7 mutant (heterozygote) exhibiting significantly elevated LDL cholesterol levels.

According to gnomAD browser, the allele frequency of APOE7 mutant is 0.002698 in East Asians, and it is not found among other ethnicities. The unexpectedly high prevalence of this variant among the phenotype of FH strongly suggests the association between this variant and LDL cholesterol.

Despite the rigorous effort of uncovering the genetic backgrounds of FH, approximately 30–40% of patients with clinical FH do not exhibit pathogenic genetic mutation(s) in established FH genes. There are several potential explanations for this situation, including so-called polygenic FH caused by the accumulation of common genetic variations elevating their LDL cholesterol. Recently, we have shown that loss-of function mutations in *ABCG5* or *ABCG8* typically regarded as the cause of recessive disorder are contributing to mimic and/or exacerbate the FH phenotype (16, 17). In this study, we extended this notion to *APOE*, at least, a rare mutant of APOE7. The clinical impact of an APOE7 mutant appears to be smaller than that of other FH mutations in *LDLR*, or *PCSK9*. However, a proportion of individuals with a mutant of APOE7 can be considered to have typical FH who are showing dominant inheritance pattern.

APOE plays an important role as the ligand, whereas the transportation of lipids mediates interaction between lipoproteins and lipoprotein receptors, including LDLR and remnant receptor. Moreover, dysfunction caused by homozygous APOE2 has been shown to cause dyslipidemia, called type III hyperlipoproteinemia (23, 24). This situation has been shown to be associated with premature ASCVD. In addition to a rather “common” situation, rare genetic variations, including a mutant APOE7, have been identified as the cause of inherited dyslipidemia. However, its prevalence and clinical impact have long been unclear. Recent advances in comprehensive genetic analytic scheme enabled us to assess this issue. We believe that the increase in triglyceride and remnant cholesterol levels is an additional risk factor for ASCVD beyond increase in LDL cholesterol level.

### Study Limitations

This study declares some limitations. First, it was an observational study assessed cross-sectionally conducted in a retrospective manner. Moreover, relatively small sample size without replication may lead to a false conclusion. Second, some patients were excluded from this analysis due to missing data, which may cause a bias. Third, validations through Sanger sequencing were not performed. Fourth, we essentially used Friedewald formula, which is closer to LDL cholesterol plus intermediate-density lipoprotein (IDL) cholesterol rather than LDL cholesterol alone. In addition, we used LDL cholesterol determined directly when triglycerides ≥400 mg/dl. The mixture of the determination may lead to biased observation. Fourth, the number of individuals with oligogenic FH was small; thus, the statistical analyses relating this group may be underpowered. Lastly, we could not account for common genetic variations associated with LDL cholesterol level in this study. Other single-nucleotide polymorphisms (SNPs) are more present and have been causally related to FH; hence, the impact of this low prevalent mutation may be small.

## Conclusions

We identified more patients with a APOE7 mutant than expected in clinically diagnosed FH patients, especially among those without FH-causing mutation. This implies that a proportion of FH cases may be caused by this particular genetic mutation.

## Data Availability Statement

Requests to access the datasets should be directed to the corresponding author.

## Ethics Statement

The studies involving human participants were reviewed and approved by IRB of the Kanazawa University. Written informed consent to participate in this study was provided by the participants' legal guardian/next of kin.

## Author Contributions

HT contributed to the design and implementation of the research, to the analysis of the results and to the writing of the manuscript. KY, NK, JS, TN, AN, SU, KS, MT, and M-aK contributed to recruiting the patients and to the writing of the manuscript. All authors contributed to the article and approved the submitted version.

## Conflict of Interest

The authors declare that the research was conducted in the absence of any commercial or financial relationships that could be construed as a potential conflict of interest.
